# A Spontaneous Coronary Artery Dissection Case Noticed during a Primary PCI

**DOI:** 10.4061/2010/794026

**Published:** 2010-03-07

**Authors:** Ismail Dogu Kilic, Halil Tanriverdi, Harun Evrengul, Sukru Gur

**Affiliations:** Department of Cardiology, Pamukkale University School of Medicine, Denizli 20070, Turkey

## Abstract

Spontaneous coronary dissections (SCAD) can be asymptomatic or can manifest itself as any of the clinical spectrum of the ischemic heart disease. In this report, we present a 65 year old man presented with myocardial infarction in whom coronary angiography a nonocclusive SCAD was noticed in addition to a very late stent thrombosis and make a brief overview of the treatment for SCAD.

## 1. Introduction

Spontaneous coronary artery dissection (SCAD) is a rarely encountered cause of acute coronary syndromes. It can manifest as any of the clinical spectrum of the ischemic heart disease such as stable angina, myocardial infarction, and sudden cardiac death. Herein, we report a case with a spontaneous dissection established during a primary percutaneous coronary angioplasty (PTCA). 

## 2. The Case

**Figure 1:**
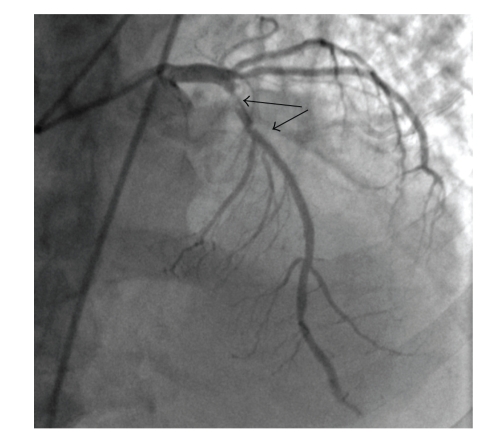
Arrows show thrombus compromising the flow in the left anterior descending artery.

**Figure 2:**
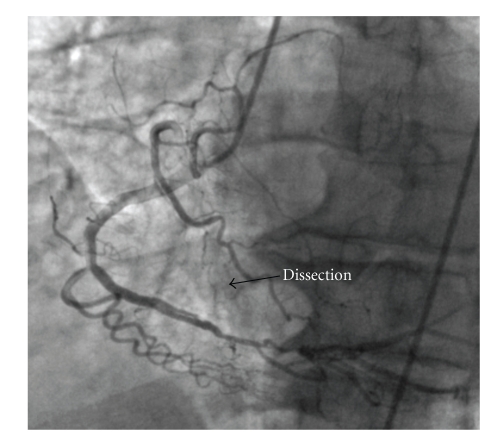
Nonocclusive right coronary artery dissection.

A 65-year-old man evaluated in the recovery room with precordial pain started 30 minutes after a surgery for spinal stenosis. ECG showed ST segment elevations in the anterior leads with inferior reciprocal ST depressions. Physical examination was unremarkable. His blood pressure was 130/90 mm Hg, and heart rate was 80 per minute. He had not experienced any anginal symptoms since he had a stent implantation 3 years ago. After the first evaluation, intravenous nitrate infusion started and the patient was taken to the catheter laboratory for a primary PTCA with the diagnosis of STEMI. In the angiography, a proximal LAD lesion (LAD, Figure 1) in the previously implanted stent region consistent with very late thrombosis and a nonocclusive spontaneous dissection in the mid portion of the right coronary artery (RCA) was observed (Figure 2). TIMI 3 flow restored chest pain and the ST elevations resolved after a successful stent implantation to the LAD. Because the dissection in the right coronary artery was not occlusive, conservative therapy was planned. IVUS could not be performed because of the technical unavailability.

Patient transferred to the coronary care unit, acetyl salicylic acid, enoxaparin, clopidogrel started under the supervision of the neurosurgery division. Laboratory tests were normal except for the elevated cardiac enzymes (with a peak troponin I value of 4.9). Echocardiography showed ejection fraction of 55%, a thickness of 13 mm of the interventricular septum, and a hypokinesia of the anterior wall of the left ventricle. Patient was discharged after 5-day uneventful follow-up. A control angiogram was performed 20 days after the initial angiogram to show a patent LAD and RCA flow despite the RCA dissection. He was symptom-free at the 3- and 6-month follow-up visits.

## 3. Discussion

Coronary artery dissection is defined as the separation of the media by hemorrhage with or without an associated intimal tear [1]. The coronary flow may be compromised by the dissection flap which in turn may cause myocardial ischemia or infarction. Spontaneous dissection of the coronary arteries is a rare cause of myocardial infarction, and only sporadic cases were reported since Pretty described the first case in 1931 [2].

There is a female predominance for SCADs with a ratio of 2: 1 [3] and SCADs are more commonly observed in the LMCA and LAD in the women in contrast to males in whom RCA dissections are more common [3] as in our case. Multivessel involvement was reported in a very small percentage of patients [4].

There is no consensus on the treatment strategies of SCAD. Conservative medical management may be an option [5, 6]. It is rational to give antiplatelet therapy because of the potential limitation of the flow caused by platelet rich thrombi [7]. Gp 2b-3a antagonist, tirofiban, was successfully used in this context [8]. However, administering thrombolytic therapy is controversial. In some series favorable results obtained with iv thrombolytic therapy given to lyse the clot in the false lumen; while in some, extension of the dissection and detoriation of the clinical status were reported [9, 10]. Koller et al. attempted to treat a case of postpartum SCAD with immunosuppressive agents (prednisone and cyclophosphamide) in addition to conventional medical therapy on the basis of observations of the eosinophilic periadventitial inflammation which suggests a role in the pathophysiology and they reported spontaneous angiographic healing of lesions [11].

Of the treatments mentioned, invasive strategies especially PTCA are more common. Although stent implantation may provide a prompt mechanical relief for the impaired coronary flow, this strategy involves the risks of coronary perforation, extending the dissection by extruding the intramural thrombus and implantation of the stent in the false lumen [7]. Good results were also achieved with coronary bypass graft surgery and are recommended as the first line treatment in cases with multivessel dissections [12]. An algorithm for the treatment of SCAD was formulated by Verma et al. [3]. Our treatment approach for the management was conservative medical treatment for the nonocclusive RCA dissection since the symptoms and ECG changes resolved. This approach confirmed to be successful by both an uneventful in-hospital follow-up and a repeat angiogram.
